# Considerations for the Nonclinical Safety Evaluation of Antibody–Drug Conjugates

**DOI:** 10.3390/antib10020015

**Published:** 2021-04-19

**Authors:** J. Edward Fisher

**Affiliations:** Center for Drug Evaluation and Research (CDER), US Food and Drug Administration (FDA), Silver Spring, MD 20993, USA; jedward.fisher@fda.hhs.gov

**Keywords:** antibody–drug conjugates, nonclinical safety studies, animal models, drivers of toxicity

## Abstract

The targeted delivery of drugs by means of linking them to antibodies (Abs) to form antibody–drug conjugates (ADCs) has become an important approach in oncology and could potentially be used in other therapeutic areas. Targeted therapy is aimed at improving clinical efficacy while minimizing adverse reactions. The nonclinical safety assessment of ADCs presents several unique challenges involving the need to examine a complex molecule, each component of which can contribute to the effects observed, in appropriate animal models. Some considerations for the nonclinical safety evaluation of ADCs based on a literature review of ADCs in clinical development (currently or previously) are discussed.

## 1. Introduction

In addition to the many therapeutic applications of unconjugated antibodies (Abs), the coupling of Abs to biologically active small molecules via chemical linkers to form antibody–drug conjugates (ADCs) has become an important strategy for increasing drug specificity [1,2]. ADCs were initially developed to increase the effectiveness of chemotherapy and reduce its toxicity by delivering cytotoxic molecules directly to tumor cells while avoiding damage to healthy cells. The majority of ADCs in clinical development combine monoclonal Abs (mAbs) specific to surface antigens present on particular tumor cells with potent anti-cancer agents for oncology indications. However, the use of ADCs as therapeutics for disease areas outside of oncology is being increasingly explored [3].

The first ADC approved by the US Food and Drug Administration (FDA), in 2000, was Mylotarg (gemtuzumab ozogamicin), a mAb to CD33 conjugated to a calicheamicin derivative for the treatment of CD33+ acute myeloid leukemia. This product was subsequently withdrawn in 2010, but then re-approved in 2017. Other ADCs approved by the FDA include, in order of approval, Adcetris (brentuximab vedotin), Kadcyla (ado-trastuzumab emtansine), Besponsa (inotuzumab ozogamicin), Polivy (polatuzumab vedotin-piiq), Padcev (enfortumab vedotin-ejfv), Enhertu (fam-trastuzumab deruxtecan-nxki), Trodelvy (sacituzumab govitecan-hziy), and Blenrep (belantamab mafodotin-blmf) [4]. Lumoxiti (moxetumomab pasudotox-tdfk), approved in 2018, is composed of only the variable domains, or antigen-binding domains, of an anti-CD22 mAb fused to a shortened form of the pseudomonas exotoxin, PE38, that inhibits protein synthesis [5]. A large number of ADCs are currently under clinical development for oncology indications that include various hematological malignancies and solid tumors as well as for targeted therapy in several autoimmune diseases, including multiple sclerosis [6].

Various strategies for the nonclinical safety evaluation of ADCs used in oncology have been described [7]. As pointed out by these authors, the complexity of ADCs requires a case-by-case scientifically based approach that relies on the application of the relevant guidelines [8,9] and close consultation with regulatory agencies [10,11]. Key determinants of the toxicity of ADCs include, in addition to antigen selection and drug mechanism of action, linker chemistry and conjugation site. For example, the development of site-specific conjugation methodologies for constructing homogeneous ADCs and more stable linkers between the drug and antibody has resulted in improved ADC safety profiles [12,13,14].

The testing strategy should take into consideration the amount of information available for each component of the ADC. An FDA review of nonclinical safety data submitted to support applications for oncology ADCs emphasized the importance of considering available information for ADCs that use the same linker and small molecule cytotoxin [10]. Many of the ADCs approved or in development for oncology indications employ the same mAb or cytotoxic agent (e.g., auristatins, maytansines, calicheamicins), some of which (e.g., trastuzumab, tanxane, methotrexate) have been approved for use alone and for which considerable nonclinical and clinical data may exist. However, the safety assessment should consider not only the amount and quality of referenceable information on the individual components but possible differences in toxicity profiles resulting from their combination in a particular ADC, such as those related to altered pharmacokinetics and tissue distribution [15,16]. The coupling of a toxin or drug to an Ab may alter the activity of either component or confer unique properties that can significantly impact the safety profile. Therefore, the most clinically relevant toxicity data would generally be expected to come from studies of the intact ADC.

## 2. Relevance of the Animal Test Species

In order for the nonclinical safety studies to adequately characterize toxicity in support of ADC clinical development, it is critical that the relevance of the animal test species and translatability of the nonclinical findings be evaluated. This determination will generally involve the examination of the binding affinity of the ADC for the target antigen in the nonclinical species relative to humans and a comparison of immunohistochemical staining profiles in cross-reactivity studies as would be typical for an unconjugated mAb [17]. Properties of the ADC resulting from the effector function or immunogenicity of the antibody may also vary across species. In addition, possible species differences in ADC toxicity associated with the other components, for example, those related to the primary pharmacology of the small molecule or linker stability, should be considered. Additionally, as with other drug and biologic products, the extrapolation of nonclinical findings to humans should include comparisons of exposure parameters and metabolism. 

Nonclinical and clinical findings for an early ADC, BR-96 doxorubicin, a chimeric mAb directed again the Lewis-Y antigen (BR96) linked to doxorubicin, illustrate some of the principles of ADC development [18]. Toxicology studies of BR96-doxorubicin, as described by Saleh et al. [19], revealed that dogs, unlike rats and monkeys, were sensitive to the toxic effects of the immunoconjugate and experienced hemorrhagic enteritis as the dose-limiting toxicity. As reviewed by Hellström et al. [20], the dog studies showed that BR96–DOX and unmodified mAb BR96 had the same dose-limiting toxicity, indicating that toxicity was mediated by the mAb, and gastrointestinal biopsies demonstrated conjugate binding to and damage of epithelial cells, most likely as a result of complement activation. According to these authors, the immunoconjugate did not induce cardiomyopathy in a rat model, unlike unconjugated doxorubicin. Consequently, dose selection for the phase I clinical trial of BR96-Dox in human subjects was based on this observation in dogs, which like humans, express the Ley antigen in epithelial cells from the gastrointestinal tract [19,20]. 

Clinical studies comparing BR-96 doxorubicin to doxorubicin alone in breast cancer patients found that the ADC was less efficacious and that toxicities were significantly different between the two treatment groups [21]. Administration of the BR96-doxorubicin conjugate was not associated with the typical side-effect profile of doxorubicin but produced gastrointestinal toxicities, including nausea, vomiting, and gastric ulceration and bleeding, as predicted by the dog studies. It was reported that one patient who received unconjugated BR96 mAb displayed as much gastrointestinal toxicity as patients receiving the corresponding dose of the conjugate, in agreement with the toxicology data in dogs [20]. Fewer hematologic toxicities were observed with the conjugate than with doxorubicin. The gastrointestinal toxicities were thought to represent the binding of the agent to normal tissues expressing the target antigen, which may have compromised the delivery of the immunoconjugate to the tumor sites [21]. Subsequent approaches to targeting Lewis antigens in the treatment of cancer have included efforts to develop mAbs with reduced cross-reactivity with normal tissues [22].

These results demonstrate the importance of identifying an appropriate test species for predicting human toxicity and the differences in toxicity profile that can be seen when a cytotoxin is targeted to specific tissues through linkage to an Ab, as well as the importance of elucidating the driver of toxicity in guiding ADC clinical development. As the following examples illustrate, each component of the ADC can be important in the determination of species relevance and the characterization of toxicity. 

## 3. Antibody Target Expression

As is the case with therapeutic mAbs, the nonclinical evaluation of ADCs may be hampered by the absence of the antigenic target in an animal test species. The ICH S6(R1) guidance on the Preclinical Safety Evaluation of Biotechnology-Derived Pharmaceuticals (2011) indicates that “Species selection for an antibody-drug/toxin conjugate (ADC) incorporating a novel toxin/toxicant should follow the same general principles as an unconjugated antibody... Relevant animal species for testing of monoclonal antibodies are those that express the desired epitope and demonstrate a similar tissue cross-reactivity profile as for human tissues [8].” Thus, species- and disease-specificity may dictate or limit the nonclinical evaluation.

Polatuzumab vedotin, which was developed to bind to CD79b on human B cells, was found not to bind to mouse, rat, or cynomolgus monkey CD79b, resulting in the lack of a pharmacologically relevant nonclinical species [23]. Nonclinical toxicity studies were conducted using a surrogate ADC that binds to cynomolgus monkey CD79b with an affinity similar to that of polatuzumab vedotin binding to human CD79b, an alternative approach to the nonclinical safety evaluation of biologics described in the ICH S6(R1) guideline [8,23]. As reported by Li et al., the surrogate ADC used the same linker–drug as polatuzumab vedotin, the microtubule inhibitor monomethyl auristatin E (MMAE) linked via the lysosomally cleavable dipeptide valine-citrulline (vc), and had a similar average number of vc-MMAE molecules conjugated to the anti-CD79b mAb (drug-to-antibody ratio, DAR), but the surrogate Ab differed from the clinical Ab in being a chimeric construct with non-humanized (mouse) complementarity determining regions. Additional studies were also performed with the human ADC to provide relevant antigen-independent pharmacology, PK, and safety information for polatuzumab vedotin [23]. The need for adequate characterization of a surrogate (e.g., with respect to epitope binding, activity and potency, and PK) for use in the safety evaluation of ADCs has been pointed out by Saber and Leighton [10]. 

Another example of an ADC with a human-specific target antigen is anetumab ravtansine, consisting of a human anti-mesothelin mAb conjugated to the maytansinoid tubulin inhibitor DM4 via a disulfide-containing linker. As described in Baumann et al. [24], on-target toxicity could not be investigated in nonclinical models because the ADC only binds human mesothelin; however, the antigen-independent, off-target toxicity of the ADC was assessed in rats and cynomolgus monkeys. It has been suggested that toxicity observed in humans may be related to the physiological expression of mesothelin in healthy tissues and that this has limited the maximum tolerated dose in clinical trials below that predicted by nonclinical studies [25]. However, the most common adverse events reported in clinical trials of anetumab ravtansine, neuropathy and keratitis, have been reported for a range of ADCs with a variety of targets [26]. While mesothelin is expressed in the normal cornea, ocular toxicities have been associated in particular with ADCs utilizing DM4 and monomethyl auristatin F (MMAF) [26,27].

Target antigen expression in normal tissues represents a major safety concern for ADC therapy. Ideally, the expression of a target antigen on normal cells should be negligible; however, as seen with the previous examples of Lewis-Y antigen and mesothelin, this may not be the case in practice. Other examples of ADC targets with the potential for toxicity related to the lack of specificity include NaPi2b, a sodium-dependent phosphate transporter expressed in several tumor types but also expressed at a detectable level in normal tissues, where it plays a role in inorganic phosphate homeostasis. An ADC (anti-NaPi2b–vc–MMAE) composed of a humanized IgG1 anti-NaPi2b mAb conjugated with MMAE through a vc peptide linker had been shown to have specific and comparable binding affinities in normal human and cynomolgus monkey tissues [28]. However, according to the report by Lin et al., despite high levels of expression in normal lung of monkeys, this cross-reactive ADC exhibited what was considered an acceptable safety profile, with a dose-limiting toxicity unrelated to normal tissue expression. In normal rats, a non-binding species, and monkeys, toxicologically significant effects were consistent with the pharmacology of MMAE, with the most sensitive tissues including the bone marrow, liver, and testes [28].

These findings were contrasted with reports of adverse dermatologic events in clinical trials of an anti-glycoprotein nonmetastatic melanoma protein B (GPNMB) mAb conjugated with MMAE, glembatumumab vedotin (CR011-vc-MMAE or CDX-011), being developed for adult cancers that express GPNMB, including melanoma and breast cancer [28,29]. GPNMB is highly expressed in a variety of tumors but also widely expressed in many normal tissues, where it is thought to be involved in regulatory roles in various cellular functions [30,31]. The highest level of expression was found in the adipose tissue and skin. It was suggested by these authors that targeting normal tissues with basal regenerative and proliferative activities with microtubule-disrupting chemotherapies presents a greater safety risk than targeting tissues, such as lung, that are not highly proliferative and thus should be less susceptible to antimitotic agents such as MMAE [28]. Although no overt CR011-vc-MMAE toxicity was reported in early nonclinical studies conducted in mice, it was acknowledged that since CR011 does not cross-react with murine GPNMB, the usefulness of this animal species for toxicity evaluation was limited [32]. The cynomolgus monkey was considered a more relevant species for toxicology studies of this ADC, since the monkey ortholog shares greater homology to human GPNMB than the rodent (approximately 95 and 70% identity at the protein level, respectively), and provided the primary basis for clinical dose selection. Additional studies examining binding affinity and tissue cross-reactivity supported the use of the monkey as an appropriate nonclinical species for the evaluation of effects on normal tissues [33,34].

However, while monkey studies correctly predicted the type of toxicity that would be observed clinically with the CD44v6-targeting immunoconjugate bivatuzumab mertansine, there appeared to be a species difference in sensitivity in studies described by Tijink et al. [35]. During the course of the nonclinical toxicity studies in cynomolgus monkeys and parallel clinical trials, it became evident that the primary toxicity of bivatuzumab mertansine involved the skin, which was thought be explained by the expression of CD44v6 on keratinocytes. While dose-related skin toxicity that was reversible and considered non-severe had been observed in the monkey toxicity studies, a fatal case of toxic epidermal necrolysis occurred in a patient with squamous cell carcinoma of the esophagus, in addition to similar less severe but unpredictable skin toxicities that resulted in a halt in development, as it was suggested that the expression of CD44v6 may not be selective enough for tumor cells [35].

## 4. Biological Activity of Drug and/or Antibody

Although the other components can contribute or modify the response, in most cases the serious or dose-limiting toxicity observed in nonclinical and clinical studies of ADCs for oncology indications has appeared to be mediated by the cytotoxic agent and/or its metabolites [26]. ADCs with various antibodies and targets have been associated with toxicities characteristic of their cytotoxin; for example, peripheral neuropathy has been seen with a number of ADCs that utilize microtubule inhibitors but most prominently with MMAE and, as previously mentioned, ocular toxicity is typically induced by ADCs that include DM4 and MMAF. The FDA analysis of nonclinical safety data for oncology ADCs found that toxicities in rodent and cynomolgus monkey in IND-enabling studies were observed mainly in the hematopoietic system, liver, and reproductive organs for all ADCs examined, and that for a given small molecule cytotoxin, the human maximum tolerated doses (MTDs) were similar regardless of the Ab target or isotype, if the linker, the DAR, and the frequency of administration remained the same [10]. This is illustrated by the overlap in toxicity profiles observed in rat and cynomolgus monkey studies of approved ADCs employing auristatins with different targets and the correspondence to effects seen with the cytotoxin alone (see Table 1).

Toxicities observed in the nonclinical studies of these ADCs were generally consistent with the expected activity of MMAE or MMAF. The observation of skin and eye toxicity in nonclinical and clinical studies of enfortumab vedotin and which are included as serious adverse reactions in Padcev product labeling were considered likely target-mediated based on Nectin-4 binding affinity and expression profiles, while the additional findings seen with belantamab mafodotin reflect known differences between the two auristatins [36,37]. Ocular toxicity associated with belantamab mafodotin was consistent with effects reported with other MMAF-conjugated ADCs [38,39]. It has been proposed that the MMAF-related inflammatory responses seen more generally may have contributed to the eye effects in animals [4]. As discussed by these authors, cytotoxin-related toxicities were often more evident in rat studies where no binding to the target occurred than in monkeys where the ADCs bound to their targets, suggesting that high levels of target expression may delay the emergence of drug-related toxicities [4,10]. The toxicities in the nonclinical studies generally correlated with clinically observed adverse reactions; however, peripheral neuropathy associated clinically with MMAE-conjugated ADCs has not been well predicted by the nonclinical studies. A more recent analysis of data submitted to the FDA to support the development of newer ADCs containing pyrrolobenzodiazepine (PBD) dimers as the cytotoxic component and with a DAR of 2 found that toxicities in animals and patients were driven mainly by the drug, with minimal or no obvious effects mediated through Ab targets, consistent with the previous conclusions [11].

The mechanisms involved in off-target ADC-related toxicity are not clearly understood, but in addition to linker-drug instability resulting in premature release of the small molecule in the plasma, it is thought that antigen-independent uptake of intact ADC can occur by both Fc-mediated and nonspecific endocytotic processes [40]. In an examination of ADC-associated ocular toxicity, Zhao et al. [41] evaluated a series of ADCs targeting ENPP3 and containing either mc-MMAF or vc-MMAE as the linker-drug. Based on the study results, which included the assessment of cytotoxicity in an in vitro assay using human corneal epithelial cells (HCECs), the authors concluded that micropinocytosis played an important role in the internalization of ADCs by HCECs and that this process is at least partly dependent on the biophysical properties of the ADC (charge and hydrophobicity), the modifications of which could impact the ADC therapeutic index. Interestingly, similar cytotoxicity in HCECs was seen between the mAb conjugated to vc-MMAE (AGS-16C3E) and that conjugated to mc-MMAF (AGS-16C3F), indicating that the effect in this assay was independent of these two linker-drug types. However, as pointed out by Mahalingaiah et al. [40], the translatability of the in vitro results reported in this study is in question since the in vivo ocular toxicity demonstrated in rabbits with these ADCs did not include damage to corneal epithelial cells but appeared to be inflammatory in nature. AGS-16C3F was reported to induce reversible ocular toxicity in patients but not produce a comparable signal in cynomolgus monkeys [41]. Although most ADCs associated with ocular toxicity contain either MMAF or DM4, ocular toxicities have also been observed for ADCs using MMAE and DM1 [42,43]. For some of these, the ocular effects may involve target expression in the eye; for example, HER2, which is targeted by trastuzumab emtansine, is reportedly expressed in human ocular surface epithelia [44]. The diverse range of ocular effects reported in association with the administration of ADCs and the variable levels of characterization of these toxicities in both nonclinical and clinical studies have contributed to the difficulty in elucidating the toxicologic mechanism(s) [45].

In the nonclinical safety assessment of trastuzumab emtansine (T-DM1), approved for the treatment of epidermal growth factor receptor 2 (HER2)-positive metastatic breast cancer, antigen-dependent and non-antigen-dependent toxicity was evaluated in cynomolgus monkeys and rats, respectively, since T-DM1 binds primate ErbB2 and human HER2 but not the rodent homolog c-neu, as reported by Poon et al. [46]. In additional studies described by these authors, rats were used to evaluate the antigen-independent effects of T-DM1 and compare them with toxicities associated with unconjugated DM1 based on previous data showing rodents to be a sensitive species for the DM1 parent compound maytansine. The majority of findings were reported to be similar for rats administered T-DM1 or DM1, and included dose-dependent effects on liver, bone marrow/hematologic systems, and lymphoid organs, although some differences in severity or type of finding were attributed to differences in pharmacokinetics, drug distribution, and/or cellular uptake between the molecules. The majority of toxicity findings in cynomolgus monkeys were reportedly similar to those seen in rats, including hepatic, bone marrow/hematologic, lymphoid organ toxicities, and testicular toxicity. However, histopathological evidence of neurotoxicity (axonal degeneration) was an additional finding in monkeys. The concordance of toxicities observed in rats and cynomolgus monkeys treated with either T-DM1 or DM1 indicated that the toxicities were primarily target-independent and consistent with the mechanism of action and pharmacologic activity of DM1 [46]. Based on the mechanism of action and other available information, the neurotoxicity observed in monkeys has also been attributed to the cytotoxic component DM1 [47]. As described in product labeling, the most frequent adverse events leading to dose reduction in patients included thrombocytopenia, increased transaminases, and peripheral neuropathy [47]. However, potentially serious cardiac toxicity has also been reported in patients treated with T-DM1. Cardiac dysfunction is a known side effect of trastuzumab, and while T-DM1-associated cardiotoxicity is not well understood, the reported clinical similarities between trastuzumab- and T-DM1-associated cardiac effects suggest that the trastuzumab component may be the driver of this toxicity [48,49].

Similar effects on the hematopoietic system and liver, including rare occurrences of sinusoidal obstruction syndrome (SOS), have been seen in patients treated with two calicheamicin conjugates (gemtuzumab ozogamicin and inotuzumab ozogamicin) targeting unrelated antigens, indicating target-independent toxicity [50]. Calicheamicin and gemtuzumab ozogamicin were both associated with hepatotoxicity in nonclinical models [51]. An off-target mechanism was further supported by studies in cynomolgus monkeys dosed with PF-0259, a nonbinding ADC containing the same linker and cytotoxin as gemtuzumab ozogamicin and inotuzumab ozogamicin, in which thrombocytopenia and microscopic liver injury consistent with early SOS were reported [52]. However, the association of serious liver toxicity with SGN-CD33A, which like gemtuzumab ozogamicin targets CD33 but employs a PBD dimer cytotoxin instead of calicheamicin, suggested that targeting CD33 may also play a role [53]. These authors stressed the importance of adequate animal models for the safety evaluation of CD33 antibody-based drugs.

As with species differences in antibody–antigen binding, there can be differences in pharmacologic and toxicologic response to a drug or cytotoxin across species. For example, in nonclinical toxicity evaluations of the cytotoxic natural product dolastatin 10, the most severe and clinically relevant effect observed was myelotoxicity, which was dose-limiting in all three species tested [54]. Myelotoxicity is an expected effect of this and related microtubule toxins, such as the synthetic auristatin derivatives, which are potent inhibitors of hematopoietic progenitor cell proliferation [55]. However, as discussed by Mirsalis et al., there were significant species differences in the dose of dolostatin 10 associated with this effect, with mice being much less sensitive than rats and dogs, which had similar MTDs [54]. Since results from in vitro bone marrow toxicity assays of granulocyte/macrophage colony formation (CFU-GM) have been shown to correlate with in vivo myelotoxicity, CFU-GM assay data from these species were compared to those from human donors in order to gain insight into which animal model more closely predicts bone marrow toxicity for humans. Following exposure to dolostatin 10, the IC90 values for myelotoxicity in CFU-GM assays were found to be similar for dogs and humans, but murine hematopoietic progenitor cells were much less sensitive than canine cells. These results indicated that dogs would be more predictive of the effects in humans. In a phase I clinical trial of dolastatin 10, granulocytopenia was dose-limiting and the human MTD was comparable to that determined in the nonclinical studies for rats and dogs. Thus, the in vitro and in vivo nonclinical results correctly predicted the human response but indicated that the mouse should not be considered an appropriate species for toxicological assessments of auristatin/dolastatin-containing ADCs [54].

The results of a toxicity study of an anti-RET mAb (Y078) conjugated to DM1 in the cynomolgus monkey, as described by Nguyen et al. [56], underscore the importance of considering all of the ADC components in study interpretation. RET, a receptor tyrosine kinase with binding sites for several signal-transducing molecules, is essential for the development of the kidney and the enteric nervous system but is also overexpressed in several cancers. As reported by these authors, Y078 was found to bind to the cynomolgus RET ortholog and to human RET with similar affinity, and tissue cross-reactivity studies showed comparable staining patterns in monkey and human tissues, indicating that the monkey was an appropriate test species for assessing on-target effects. The toxicity study found that both Y078-DM1 and, to a lesser degree, unconjugated Y078 were associated with dose-dependent peripheral neuropathy. Since RET is expressed in the adult peripheral nervous system, this was an expected outcome of the targeted delivery of a potent microtubule-interfering drug to cells expressing RET. However, the neurotoxicity seen with unconjugated Y078 indicated a contribution of the Ab in addition to that of targeted delivery [56].

Unexpected toxicity thought to be due to the antibody component of the ADC rather than the small molecule was reported with MEDI-547, an ADC composed of a mAb directed against the receptor tyrosine kinase EphA2 conjugated with MMAF that has been investigated for the treatment of solid tumors [57]. As described by Annunziata et al., MEDI-547 was found to bind human, cynomolgus monkey, mouse, and rat EphA2 with similar affinities via the highly conserved extracellular domain. However, while clotting abnormalities reported in patients in a phase 1 study showed similarities to those seen in the nonclinical studies in all three species, serious hematological toxicity occurred in humans at doses much lower than those predicted to be safe based on the nonclinical studies; 1/10 the highest non-severely toxic dose in cynomolgus monkeys exceeded the MTD in patients, leading to the discontinuation of the clinical investigation. Based on pharmacokinetic data indicating minimal or no dissociation of the conjugate and experience with other auristatin-containing ADCs, the authors concluded that the Ab was likely responsible for the toxicity observed in the study [57].

## 5. Linker Chemistry

Linker chemistry is an important determinant of the safety and efficacy of ADCs. Linkers, which are generally divided into cleavable and non-cleavable types, are designed to be stable in the bloodstream while allowing efficient release of the small molecule cytotoxin after internalization in the target cell [58]. The stability of antibody–drug linkers in systemic circulation has been a key focus in safety evaluations, since premature loss of the small molecule prior to ADC target cell internalization can result in off-target toxicity from non-specific drug release [59]. Strategies to develop ADCs with more stable linkers and lower levels of unconjugated mAbs have been viewed as critical to the effort to improve ADC pharmacokinetic properties, therapeutic indexes, and safety profiles [13,60].

An insufficiently stable linker was thought to represent a possible liability for Mylotarg, although subsequent ADCs utilizing similar acid-labile hydrazone linkers, e.g., inotuzumab ozogamicin and milatuzumab-doxorubicin, have shown good stability in human plasma and serum [14]. However, a comparison of ADCs containing hydrazone linkers with those using protease-sensitive dipeptide linkers showed that the peptide-linked conjugates were much more stable in buffers and plasma than the corresponding hydrazone conjugates, and as a result exhibited more specific delivery and lower systemic toxicity [61]. These results illustrated the importance of conjugation and linker chemistry in achieving a favorable therapeutic index.

Species differences in drug-linker stability can lead to difficulties in evaluating the therapeutic index of an ADC based on nonclinical studies. In studies examining linker modifications that could make nonclinical studies of ADCs utilizing the cleavable valine-citrulline-p-aminobenzylcarbamate (VC-PABC) linker more comparable among species by decreasing exposure differences caused by interspecies differences in linker cleavage, the enzymatic VC-PABC cleavage observed in mouse, the species most commonly used in efficacy studies, was contrasted with the lower degree of instability detected in the plasma of rat and cynomolgus monkey, the species generally used for toxicity studies [62]. These differences were attributed to species differences in carboxylesterase activity and substrate specificity. In a report describing an in vitro whole blood assay to predict in vivo stability, cross-species differences for a variety of ADCs were highlighted and thought to reflect differences in enzyme expression and activity [63].

Linkers can also affect the physicochemical properties of the ADC, such as the tendency to aggregate, which can impact ADC efficacy, hepatotoxicity, and immunogenicity [64,65]. When hydrophilic glucuronide-based drug linkers were compared to dipeptide linkers during the development of ADCs employing camptothecin cytotoxins, the more hydrophilic glucuronide drug linkers led to less antibody aggregation than the dipeptide drug linkers [66]. This study also reported that the dipeptide (vc) conjugate of a camptothecin analog with a mAb against the Lewis-Y antigen had no effect on tumor growth in a mouse model, while the corresponding glucuronide conjugate induced substantial tumor growth delay.

## 6. Conclusions

The goal of nonclinical toxicology studies is to predict potential safety risks in humans. The predictive value of nonclinical safety studies of ADCs can be enhanced by the appropriate choice of animal test species and knowledge of the mechanism by which adverse effects are produced or which attributes of the ADC drive the observed toxic effect. Some of the factors which influence toxic responses to ADCs and the translatability of nonclinical toxicology results to clinical use, including those involved in interspecies differences in toxic responses, are discussed in this review. Each ADC component, i.e., antibody, linker, and small molecule, needs be considered when selecting the appropriate test species and interpreting study results, which adds to the complexity of evaluating ADC safety in nonclinical studies.

In an examination of possible reasons for the failure of animal studies to predict the peripheral neuropathy seen clinically with the vc-MMAE ADC platform, several possibilities were hypothesized and evaluated in detail [67]. After considering possible roles of antibody, drug, and linker, the authors concluded that differences in dosing regimen and the resulting differences in the level of and duration of exposure to MMAE in the peripheral nerve tissue are the most likely explanation. These pharmacokinetic parameters provide a critical basis for animal to human comparisons for any therapeutic, not specifically in relation to ADC use. However, the systematic manner of evaluation performed for this linker-drug is nonetheless instructive. This assessment included the consideration of possible species differences in antigen expression, type and stability of linker, and properties of the cytotoxin. These and other authors have also emphasized the importance of using sensitive techniques (in, e.g., neurotoxicity and ocular toxicity assessments) in appropriate nonclinical species in order to improve the predictive capability of the animal model.

Examples of nonclinical and clinical toxicity results reported during the clinical development of ADCs for oncology indications demonstrate that each component of the ADC can play a role in the observed effects and should be considered when designing and interpreting the nonclinical safety studies. Although serious or dose-limiting toxicity appeared to be primarily mediated by the small molecule in most cases and the effects seen characteristic of the cytotoxic drugs used in the treatment of cancer independent of target, different patterns may emerge as the field progresses and expands to include ADCs for use in other therapeutic areas. The general principles discussed should be applicable to many types of toxicology studies, the need for which will depend on the safety and regulatory requirements for a specific ADC as determined by factors such as clinical indication.

## Figures and Tables

**Table 1:** Toxicities observed in nonclinical studies and human adverse reactions reported in labeling for approved auristatin-conjugated ADCs ^1^.

ADC	Target	Linker-Drug	Rat	Target Binding	Cynomolgus Monkey	Target Binding	Human SARs ^2^
Adcetris (brentuximab vedotin)	CD30	vc-MMAE	Hematopoietic system, liver, male reproductive organs	no	Hematopoietic system	yes	Peripheral neuropathy, hematologic toxicities, hepatotoxicity
Polivy (polatuzumab vedotin)	CD79b	vc-MMAE	Hematopoietic system, liver, male reproductive organs	no	Hematopoietic system	yes	Peripheral neuropathy, myelosuppression, hepatotoxicity
Padcev (enfortumab vedotin)	Nectin-4	vc-MMAE	Hematopoietic system, liver, reproductive organs, skin, eye ^3^	yes	Hematopoietic system, liver, GI tract, skin, eye ^3^	yes	Peripheral neuropathy, ocular disorders, skin reactions
Blenrep (belantamab mafodotin)	BCMA	mc-MMAF	Hematopoietic system, liver, kidney, lung, reproductive organs, eye ^4^	no	Hematopoietic system, liver, kidney, lung, reproductive organs	yes	Ocular toxicity, thrombocytopenia

ADCs, antibody–drug conjugates. ^1^ Information from publicly available FDA reviews and product labeling. ^2^ Serious adverse reactions described in the product label. ^3^ Eye toxicity consisted of increased mitotic figures in the corneal epithelium in rat and histopathological findings of lymphocyte infiltration in the lacrimal gland accompanied by observations of ptosis, eye discharge, and periorbital swelling in monkey. There were no significant findings in the in-life ophthalmologic examinations (slit lamp) in rat and monkey. ^4^ Increased mitoses of corneal epithelial cells with bilateral single-cell necrosis were observed in rats and rabbits.

## Data Availability

Not applicable.
